# Leucovorin rescue allows effective high-dose pralatrexate treatment and an increase in therapeutic index in mesothelioma xenografts

**DOI:** 10.1007/s00280-014-2580-z

**Published:** 2014-09-09

**Authors:** Philip M. Tedeschi, Yamini K. Kathari, Iqra N. Farooqi, Joseph R. Bertino

**Affiliations:** Departments of Pharmacology and Medicine, Rutgers Cancer Institute of New Jersey, Rutgers, The State University of New Jersey, 195 Little Albany Street, New Brunswick, NJ USA

**Keywords:** Pralatrexate, Antifolate, Mesothelioma, Leucovorin, Folinic acid

## Abstract

**Purpose:**

To investigate the ability of leucovorin (LV) to abrogate dose-limiting toxicities of pralatrexate (PDX) while maintaining efficacy, in vivo.

**Methods:**

H2052 mesothelioma cells were treated with the antifolates methotrexate (MTX), PDX and pemetrexed, with and without LV rescue 24 h later. Cell killing was evaluated 48 h later. Female nude mice bearing H2052 xenografts were treated with varying doses and schedules of the antifolate PDX and LV.

**Results:**

In vitro, H2052 cells were more sensitive to PDX as compared to MTX and pemetrexed. Administration of LV 24 h after antifolate treatment reduced efficacy of antifolates MTX and pemetrexed, but not PDX. In vivo, LV was found to reduce toxicity of PDX at the maximum tolerated dose without sacrificing efficacy. Lethal doses of PDX were rescued by LV, and mice bearing the H2052 tumor demonstrated prolonged and enhanced tumor regression.

**Conclusions:**

High-dose PDX with subsequent LV rescue may be a viable treatment strategy in mesothelioma and other cancers. The inclusion of LV rescue into new and existing PDX treatment protocols should be explored as a way to expand the tolerability and effectiveness of PDX in the clinic.

## Introduction

Folic acid and derivatives (folates) are required by mammalian cells to carry out one-carbon transfer reactions and the de novo synthesis of nucleic acids [1]. Folate cannot be synthesized de novo and must be consumed. Once internalized, folic acid is reduced by dihydrofolate reductase (DHFR) to tetrahydrofolate, which then can act to deliver one carbon units to acceptor molecules. Antifolates target rapidly cycling cancer cells, whose enhanced need of one carbon units for de novo pyrimidine and purine synthesis leaves them vulnerable to DHFR inhibition [2]. Aminopterin, a DHFR inhibitor, was the first antifolate used in the clinic, but it fell out of favor when less toxic methotrexate (MTX) was introduced in the 1950s [3]. MTX continues to be included in many therapeutic regimens today.

Pralatrexate (PDX) is a second generation antifolate recently approved for the treatment of peripheral T-cell lymphoma [4]. In addition to inhibiting DHFR, PDX also is an excellent substrate for folylpolyglutamate synthase (FPGS) which polyglutamylates folates, leading to greater cellular retention and activity, and the reduced folate carrier-1 (RFC-1), the primary folate membrane transporter [5]. These additional PDX properties that increase PDX transport and retention in cancer cells lead to improved efficacy when compared to MTX [6].

**Fig. 1:**
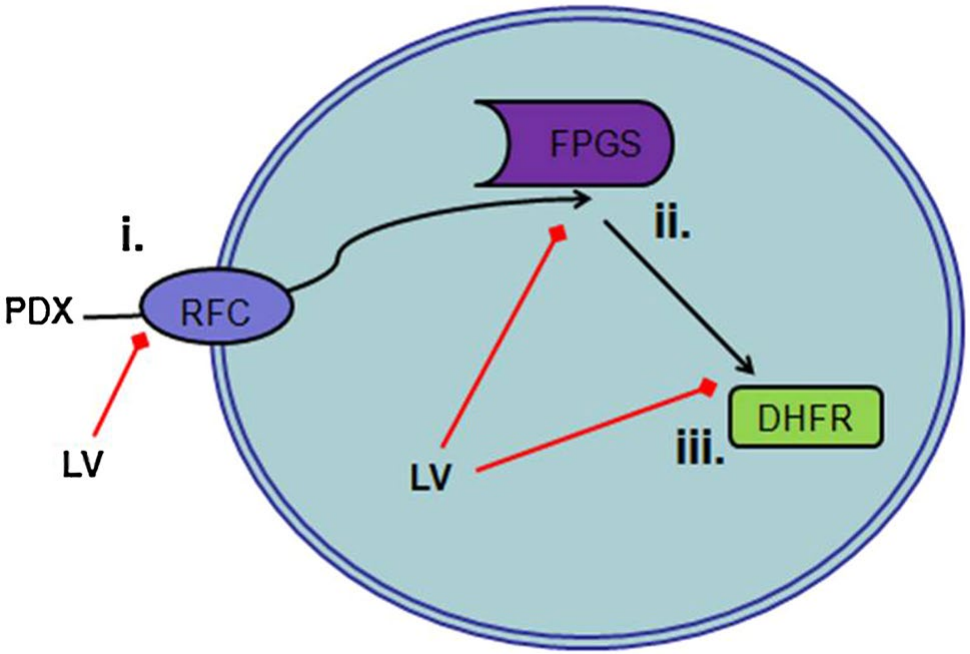
Leucovorin (LV) may abrogate pralatrexate (PDX) activity through three mechanisms. *i* Competition for reduced folate carrier type 1 (RFC) transport into cell. *ii* Competition for polyglutamylation, a retention and activity marker, by folylpolyglutamate synthase (FPGS). *iii* Provides an alternate source of tetrahydrofolate, working around PDX inhibition of dihydrofolate reductase (DHFR)

Adverse events associated with antifolates can be severe, commonly mucositis and leukopenia. A strategy to avoid toxicity and still provide clinical benefit is the inclusion of leucovorin (LV) in high-dose MTX regimens [7]. LV is a stable, reduced form of folate that is converted to tetrahydrofolate without requiring DHFR, thus bypassing the inhibition of tetrahydrofolate synthesis by MTX and PDX. LV also competes for binding with antifolates at the reduced folate carrier, and when converted to tetrahydrofolate, competes with antifolates for polyglutamylation (Fig. 1).

In this study, we demonstrate the protective effect of LV on PDX toxicity and show that a significant antitumor effect is achievable with high-dose PDX and LV rescue in mesothelioma xenografts.

## Materials and methods

### In vitro cytotoxicity

Five thousand H2052 pleural mesothelioma cells per well were plated in 96-well plates in RPMI 1640 media (Gibco) supplemented with 10 % dialyzed FBS (Invitrogen). The following day, spent media was removed and fresh media containing drug was added and plates were incubated for 24 h. Drug containing media was removed and media containing 2μM LV was added and plates were incubated for 24 h. LV containing media was removed and fresh media was added to the plates incubated for 48 h. The Cell Titer 96 Aqueous One Solution (Promega) assay was used to assess cell viability at the end of the experiment according to manufacturers’ protocol. Data were analyzed using the GraphPad Prism 6 software package (GraphPad Software Inc.).

### In vivo experiments

Eight- to 10-week-old female nude mice (Taconic) were subcutaneously injected on the right flank with 5 million H2052 cells in a 1:1 mixture of matrigel (BD Biosciences) and PBS (Gibco). Mice were monitored until palpable tumors developed and reached a size of 100 mm^3^. Mice were then randomized and split into the following treatment groups: saline control; 60 or 180 mg/kg PDX on days 1, 4 and 7; 60 or 180 mg/kg PDX on days 1, 4 and 7 followed by 50 mg/kg LV 24, 32 and 48 h after each PDX administration; 50 mg/kg LV following the above schedule. General toxicity was monitored through weight loss. Tumor dimensions were measured every 2–4 days using calipers, and tumor volume was calculated using the following equation: Volume = (width^2^) × (length/2). All animal experiments were approved by the Rutgers Institutional Animal Care and Use Committee.

## Results

**Fig. 2:**
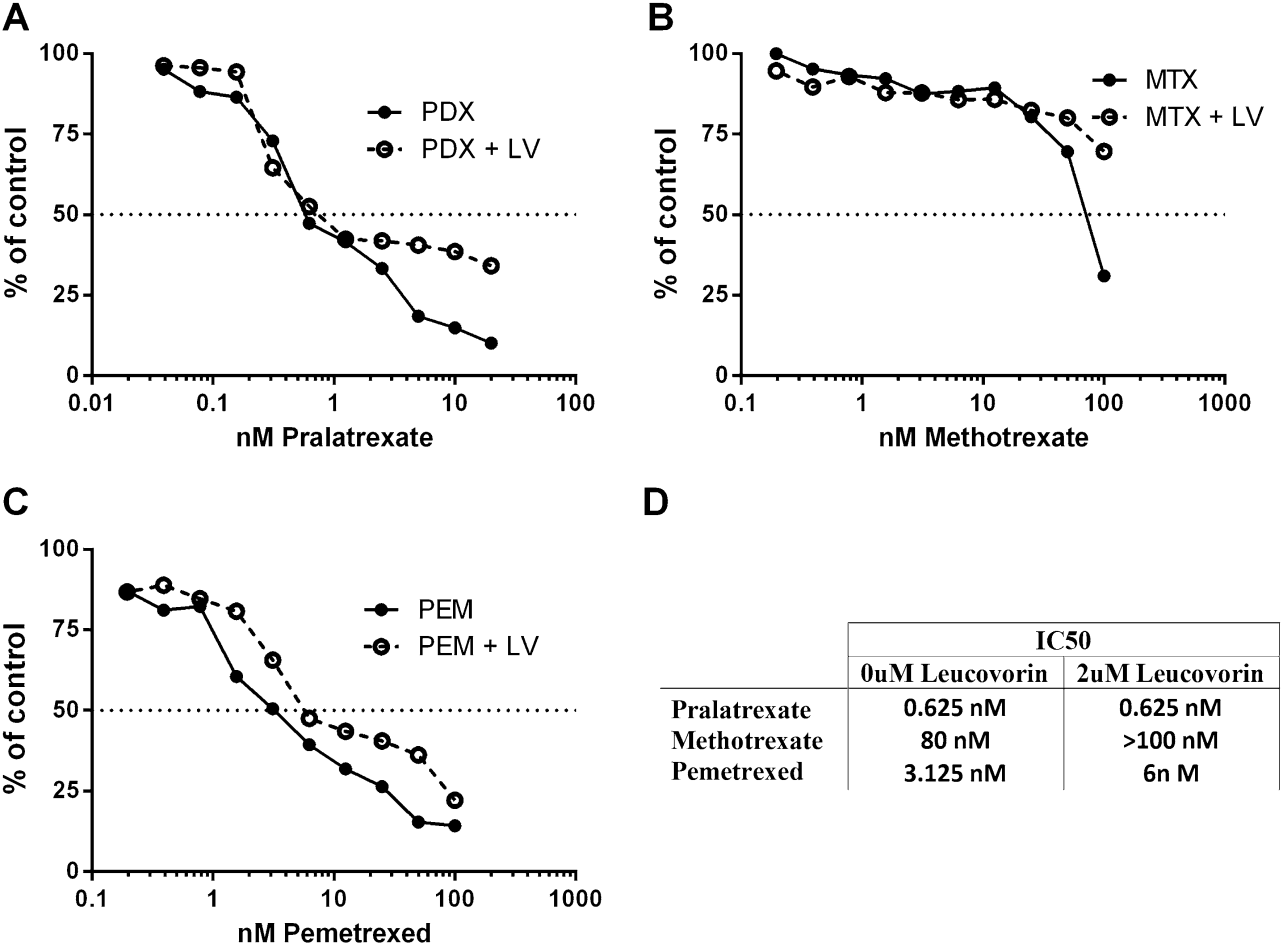
Pralatrexate (PDX) is more potent than methotrexate and pemetrexed in vitro. Dose response curves shown as percent of viable H2052 mesothelioma cells remaining after 24 h treatment with pralatrexate (**a**), methotrexate (**b**) or pemetrexed (**c**) followed by 72 h culture in fresh media (*solid line*) or followed by 24 h culture in 2μM leucovorin and 48 h culture in media (*dashed line*). **d** A summary table of IC50 values

To assess the sensitivity to antifolate therapy, a comparison of MTX, PDX and pemetrexed in the pleural mesothelioma cell line H2052 was performed. After a 24 h treatment with drug, the media was removed and replaced with fresh media and cultured a further 72 h. PDX demonstrated significant efficacy over MTX in this assay, with IC_50_ values of 0.625 and 80 nM, respectively (Fig. 2a, b). The large difference between PDX and MTX is likely due to enhanced transport and retention of PDX. H2052 cells were also more sensitive to PDX than pemetrexed (Fig. 2c), an antifolate currently used in the clinic to treat mesothelioma.

As LV can abrogate antifolate toxicity, we next examined if efficacy of PDX would be compromised by the addition of LV 24 h later. H2052 cells were treated with antifolate as before, but media containing 2μM LV was added for another 24 h period, followed by removal of drugs and culture in fresh media for a further 48 h. The addition of 2μM LV raised the IC_50_ slightly for MTX and pemetrexed (Fig. 2d). The effect of LV on tumor cell sensitivity to PDX was only at high PDX concentrations, and was not expected to be significant in in vivo xenografts. We predicted that using PDX in combination with LV rescue would result in an increased therapeutic index.

**Fig. 3:**
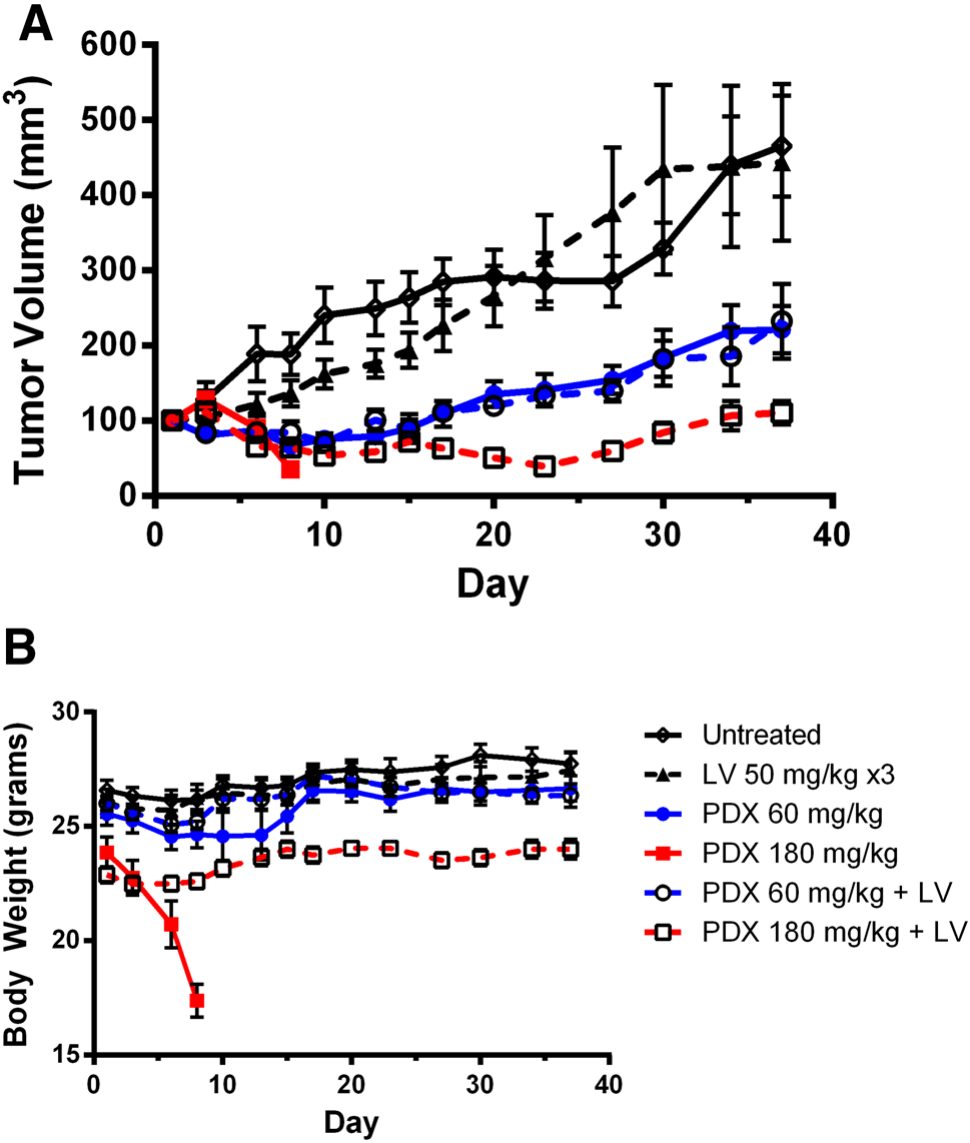
A and B Pralatrexate is effective against H2052 xenografts. Mice bearing 100 mm^3^ tumors were treated by i.p injection of saline control; 60 or 180 mg/kg PDX on days 1, 4 and 7; 60 or 180 mg/kg PDX on days 1, 4 and 7 followed by 50 mg/kg leucovorin 24, 32 and 48 h after each PDX administration; 50 mg/kg leucovorin. Tumor volume (**a**) and body weight (**b**) were measured

The effect of LV treatment on PDX efficacy and toxicity was examined using H2052 xenografts in nude mice (Fig. 3). Using 60 mg/kg as the equivalent clinical dose, tumor regression was seen both with and without LV treatment in this cohort. Efficacy was nearly identical, but mice that were treated with LV had less toxicity and bodyweight remained close to control animals.

To mimic high-dose MTX treatment with LV rescue, using PDX, we included a cohort treated at 180 mg/kg PDX with and without LV rescue. Without LV rescue, mice lost a large amount of weight, had poor BCS scores and had to be killed according to institutional protocol (Fig. 3b). Mice rescued with LV exhibited some weight loss, and a durable response lasting several weeks was observed (Fig. 3a).

## Discussion

Surgical resection followed by chemotherapy is the preferred method of treatment for malignant mesothelioma [9]. The antifolate pemetrexed is currently used alone and in combination with cisplatin in treating mesothelioma, with modest response rates seen in the clinic [10]. Chemotherapy is primarily palliative in mesothelioma and there is a need for new and innovative therapies that have the ability to improve patient outcomes [11].

There were no responses to PDX in a phase II study in mesothelioma [12]. To improve tolerability, PDX was administered at 135 mg/m^3^ biweekly instead of weekly, possibly limiting clinical effectiveness. A dose schedule of PDX weekly with LV rescue may demonstrate improved antitumor activity with tolerable toxicity. Supporting this conclusion, a small recent case series exploring LV rescue and PDX in T-cell cutaneous lymphoma found that LV rescue allowed patients to tolerate PDX therapy while preserving efficacy [8]. Dose scheduling must be optimized in patients, as the interval between antifolate and LV administration has recently been shown to be significant in determining the efficacy of therapy in vitro [13].

The incorporation of LV rescue with current PDX dosing regimens may prove an effective method to prevent DLTs and increase the therapeutic index. While a phase II study of PDX in patients with mesothelioma did not show responses, higher doses with the inclusion of LV rescue into new and existing PDX treatment protocols should be explored as a way to expand the tolerability and effectiveness of PDX in the clinic.
